# Tumor Microenvironment Responsive Pepper Mild Mottle Virus-Based Nanotubes for Targeted Delivery and Controlled Release of Paclitaxel

**DOI:** 10.3389/fbioe.2021.763661

**Published:** 2021-09-30

**Authors:** Jiejun Peng, Yueyan Yin, Hongze Liang, Yuwen Lu, Hongying Zheng, Guanwei Wu, Shaofei Rao, Jianping Chen, Fei Yan, Jingbo Hu

**Affiliations:** ^1^ State Key Laboratory for Managing Biotic and Chemical Threats to the Quality and Safety of Agroproducts, Institute of Plant Virology, Ningbo University, Ningbo, China; ^2^ College of Plant Protection, Yunnan Agricultural University, Kunming, China; ^3^ Institute of Alpine Economic Plants, Yunnan Academy of Agricultural Sciences, Lijiang, China; ^4^ Faculty of Materials Science and Chemical Engineering, Ningbo University, Ningbo, China

**Keywords:** pepper mild mottle virus, folate, nanotubes, pH-responsive, paclitaxel

## Abstract

Plant virus nanoparticles (PVNPs) have been widely used for drug delivery, antibody development and medical imaging because of their good biodegradation and biocompatibility. Particles of pepper mild mottle virus (PMMoV) are elongated and may be useful as drug carriers because their shape favours long circulation, preferential distribution and increased cellular uptake. Moreover, its effective degradation in an acidic microenvironment enables a pH-responsive release of the encapsulated drug. In this study, genetic engineering techniques were used to form rod-shaped structures of nanoparticles (PMMoV) and folated-modified PMMoV nanotubes were prepared by polyethylene glycol (PEG) to provide targeted delivery of paclitaxel (PTX). FA@PMMoV@PTX nanotubes were designed to selectively target tumor cells and to release the encapsulated PTX in response to pH. Efficient cell uptake of FA@PMMoV@PTX nanotubes was observed when incubated with tumor cells, and FA@PMMoV@PTX nanotubes had superior cytotoxicity to free PTX, as reflected by cell survival and apoptosis. This system is a strong candidate for use in developing improved strategies for targeted treatment of tumors.

## Introduction

Cancer is one of the most common causes of death in humans. Each year there are about 10 million new cancer cases worldwide, and more than seven million people die from the disease (Bedard et al., 2020). In China, more than 1.6 million people die of cancer each year, making it the single greatest cause of death. Tumor chemotherapy remains the classic, and most commonly used, cancer treatment but problems are encountered in the deployment of most chemotherapeutic drugs. These problems include poor solubility, non-selective distribution in the human body, and drug resistance, all of which weaken the therapeutic outcomes.

Paclitaxel (PTX) is a well-known broad-spectrum anti-tumor agent. It induces and promotes both tubulin polymerization and microtubule assembly while preventing depolymerization, thereby stabilizing microtubules and inhibiting cancer cell mitosis and triggering apoptosis (Pham et al., 2020). Its direct adverse effects include bone marrow suppression, while the addition of castor oil to increase its water solubility can also cause severe nephrotoxicity and neurotoxicity. To improve clinical efficacy and reduce side effects, better drug delivery systems are needed to increase the bioavailability of anti-tumor agents and their targeted distribution to tumor tissues.

Plant viruses occur widely in nature and have a variety of particle morphologies, including rod-shaped, filamentous, bullet-shaped and spherical. Most of them are not covered by membranes and their particles are relatively stable. Plant virus nanoparticles are beginning to be used for nano-drug delivery, antibody preparation, and medical imaging because of their biodegradability, biocompatibility, and monodisperse nanoparticles (Wen and Steinmetz, 2016). The most commonly used plant virus vectors to date include tobacco mosaic virus, potato virus X and pea mosaic virus.

Pepper mild mottle virus (PMMoV) belongs to the genus *Tobamovirus* and has a positive-strand RNA genome (Rodríguez-Cerezo et al., 1989; Alonso et al., 1991). The virus infects various solanaceous plants worldwide, and there have been several recent reports that it is universally present in human feces and can be used as an indicator of human fecal pollution of water (Hamza et al., 2011; Peng et al., 2015; Symonds et al., 2019). The genome of PMMoV has 6,356–6,357 nucleotides (nts) and encodes at least four open reading frames (ORFs), including the 126 kDa and read-through 183 kDa RNA-dependent RNA polymerase (RdRp) protein, a 30 kDa movement protein (MP) and a 17 kDa coat protein (CP) (Avila-Rincon et al., 1989; Alonso et al., 1991; Velasco et al., 2002). Virus-infected pepper plants have abundant tobamovirus-like particles which are rod-shaped, measuring 300 × 18 nm with a 4 nm central longitudinal cavity (Peng et al., 2015). This linear morphology can promote long circulation, tumor homing, tumor penetration and cell uptake.

Here we describe the preparation of rod-shaped PMMoV nanoparticles by genetic engineering of an infectious virus clone in the laboratory host plant *Nicotiana benthamiana.* Purified virions were then loaded with different drug combinations. The tumor targeting molecule folate-decorated PEG (FA-PEG) and PEG were used to coprecipitate PMMoV, and PTX was incorporated into the interior cavity of the virus particles. The performance of FA@PMMoV nanotubes as a drug delivery carrier, including factors such as drug-loading, drug release, internalization and *in vitro* anti-tumor activity, were then investigated in detail.

## Results and Discussion

### Purification of Rod-Shaped Nanoparticles in *N. benthamiana* by Infection With Full-Length cDNA Clone

**FIGURE 1:**
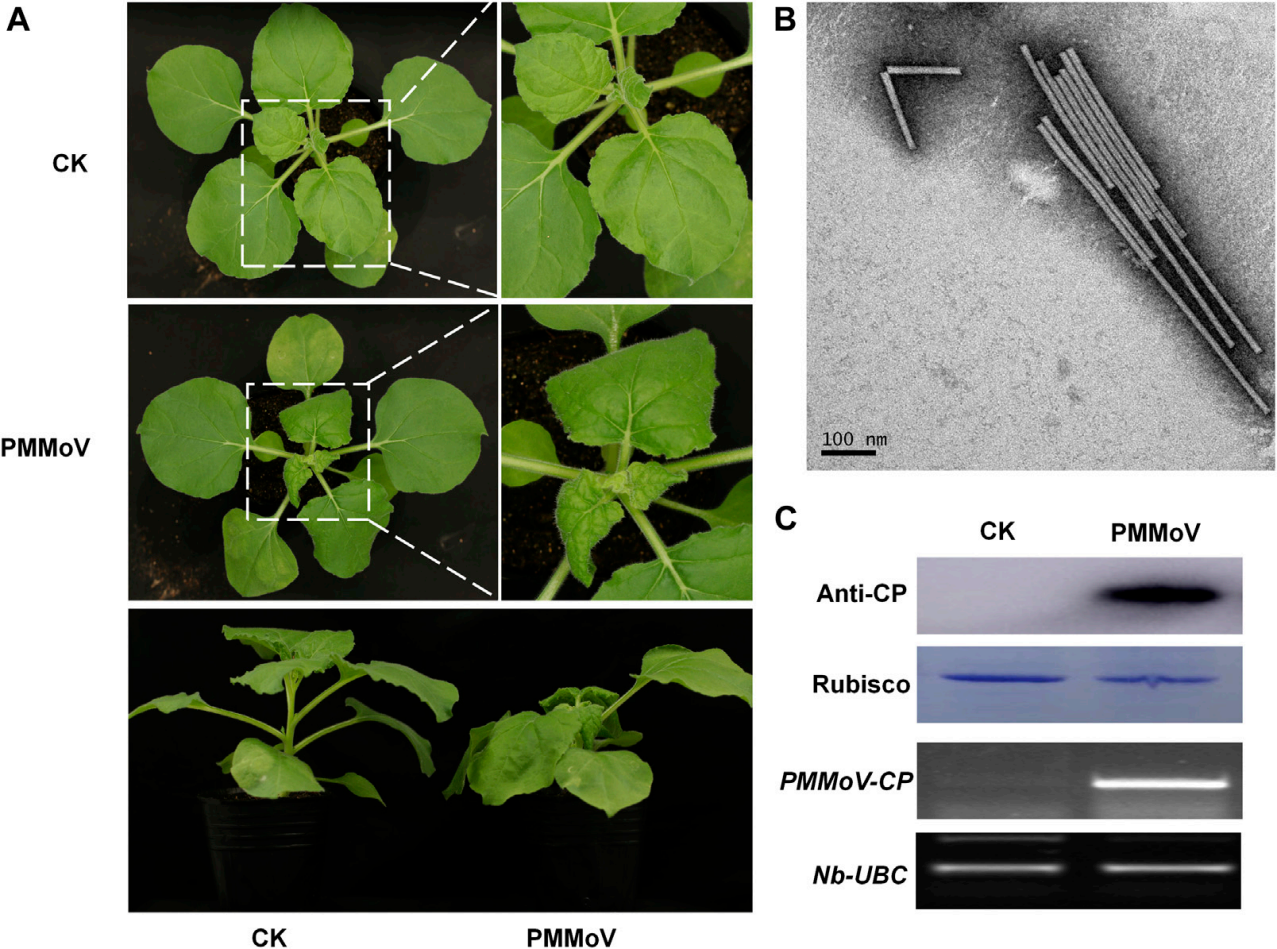
Symptoms caused by PMMoV in *N. benthamiana* infected with a full-length cDNA infectious clone. **(A)** Phenotype of *N. benthamiana* plants agroinfiltrated with viral infectious clone combinations or empty agrobacterium (CK) at 28 days post infiltration. **(B)** Virions purified from leaves infected by the PMMoV infectious clones and observed by TEM. Bars represent 100 nm. **(C)** Detection of PMMoV CP by Western blot (upper panels) and RT-PCR confirming the presence of viral RNAs (lower panels) in systemic leaves of inoculated plants. Rubisco and Nb-UBC are the respective loading controls.

To obtain the rod-shaped nanoparticles, a genetically engineered vector of PMMoV was inoculated to *Nicotiana benthamiana* by infiltrating its leaves with *Agrobacterium tumefaciens*. Typical viral symptoms had developed in PMMoV-infected plants 14 days post-inoculation (dpi) (Figure 1A). Both RT-PCR and Western blot showed that PMMoV had spread systemically in the inoculated plants (Figure 1C). After collecting the systemically-infected leaves and extracting the virions, transmission electron microscopy (TEM) showed the presence of rod-shaped nanoparticles in the sucrose-purified suspension (Figure 1B). Using UV absorbance at 280 nm, it was estimated that yield was about 1 g of virions per 100 g virus-infected leaves.

### Characterization of FA@PMMoV@PTX Nanotubes

The chemical structure of FA-PEG was confirmed by hydrogen-nuclear magnetic resonance spectroscopy (^*1*^ *H* NMR), as shown in Supplementary Figure 1. The typical peaks at 4.51 (a), 6.60 (b), 7.68 (c) and 8.67 (d) ppm representing the aromatic and pteridine protons of FA were detected in FA-PEG, suggesting that FA was successfully conjugated to PEG.

The content of PTX in FA@PMMoV@PTX nanotubes was determined by high performance liquid chromatograph (HPLC). PTX in FA@PMMoV@PTX nanotubes was extracted with methanol according to the principle of similar polarity, standard curve formula, A = 27.594×C-18.255 (*R* ^2^ = 0.9995). In the concentration range of 0.5–100 μg/ml, the chromatographic peak area A measured by the HPLC method has a good linear relationship with the drug concentration C. The drug loading of PTX in FA@PMMoV@PTX nanotubes was 1.78 ± 0.34% at 5% feeding ratio. The particle size of FA@PMMoV@PTX nanotubes was 357.7 and 56.4 nm respectively, which was associated with its high aspect ratio. In addition, its zeta potential was -23.5 ± 7.3 mV.

### Stability of FA@PMMoV@PTX Nanotubes

**FIGURE 2:**
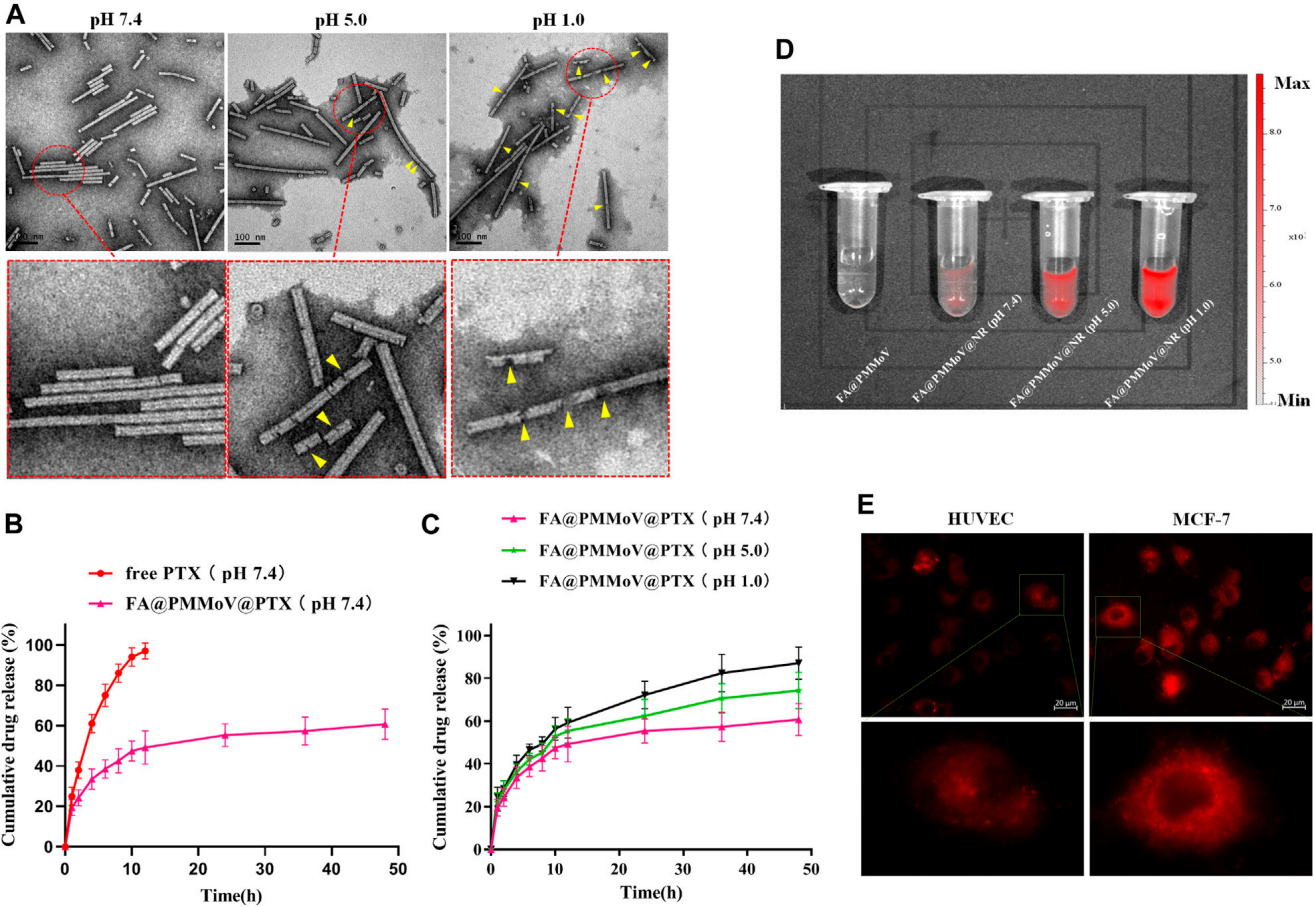
Characterization of FA@PMMoV@PTX nanotubes. **(A)** TEM images of FA@PMMoV@PTX nanotubes after incubation in media at different pH values. **(B)** The *in vitro* drug release of FA@PMMoV@PTX nanotubes in pH 7.4 medium, using free PTX as a control. **(C)** The *in vitro* drug release of FA@PMMoV@PTX nanotubes in media at pH 7.4, 5.0, and 1.0. **(D)** Images showing the fluorescence of FA@PMMoV@NR nanotubes after incubation in media at different pH values. (E) Images showing the fluorescence of FA@PMMoV@Dir nanotubes in MCF-7 cells and HUVEC (controls).

The stability of FA@PMMoV@PTX nanotubes under different pH condition was investigated using TEM. FA@PMMoV@PTX nanotubes were dispersed in PBS with different pH values (1.0, 5.0 and 7.4) and then incubated for 24 h. After that, the solution was collected, sprayed on copper mesh and observed by transmission electron microscopy (TEM). As shown in Figure 2A, FA@PMMoV@PTX nanotubes were stable at pH 7.4 but were obviously degraded to some extent after incubation at pH 5.0 and more severely at pH 1.0 (yellow arrows in the partially enlarged pictures).

### pH-Triggered Release of FA@PMMoV@PTX Nanotubes

It seemed possible that the pH-responsive degradation of FA@PMMoV@PTX nanotubes might lead to leakage of PTX from the interior cavity of FA@PMMoV nanotubes. We therefore investigated the pH-triggered release behavior of FA@PMMoV@PTX nanotubes in release media of different pHs, using free PTX as a control. The solubility of PTX in water is extremely low (0.3 μg/ml at 37°C). In order to ensure the sink conditions in the *in vitro* release experiment, PBS containing 1 M sodium salicylate was used as the release medium (Hu et al., 2015). As shown in Figure 2B, the release of PTX from FA@PMMoV@PTX nanotubes at pH 7.4 was slower than in the control. Interestingly, the release of PTX from FA@PMMoV@PTX nanotubes obviously accelerated in the more acidic media (pH 1.0 and 5.0; Figure 2C), and so the release was positively associated with pH-dependent degradation of the FA@PMMoV@PTX nanotubes.

The pH-triggered release behavior of FA@PMMoV nanotubes was further confirmed by Nile Red (NR)-based fluorescence. The changes of the NR-based fluorescent signal represent the release of NR from FA@PMMoV@NR nanotubes, and this was detected using the *In-Vivo* imaging system (PerkinElmer, IVIS Lumia III). The fluorescence of NR is low when encapsulated in the nanocarrier, but strong fluorescence occurs after release from the nanocarrier. As shown in Figure 2D, the release of NR from FA@PMMoV@NR nanotubes was pH-dependent with stronger signals in medium at pH 1.0 than at pH 7.4.

Intracellular pH-responsive drug release of FA@PMMoV@NR nanotubes was also examined by detecting the NR fluorescence signal in tumors (MCF-7) and human umbilical vein endothelial cells (HUVEC), visualized under an inverted fluorescence microscope (Zeiss, Axio Observer 5). As shown in Figure 2E, the intracellular NR fluorescent signal in MCF-7 cells was obviously higher than that in HUVEC, which demonstrated that FA@PMMoV nanotubes could achieve faster drug release in the acidic environment due to the degradation of FA@PMMoV@NR nanotubes in tumor cells. Thus, the pH-responsive drug release of FA@PMMoV nanotubes contributed to the selective release of the anti-tumor agent, and this potentially increased the safety of anti-tumor therapy.

### Cellular Uptake

**FIGURE 3:**
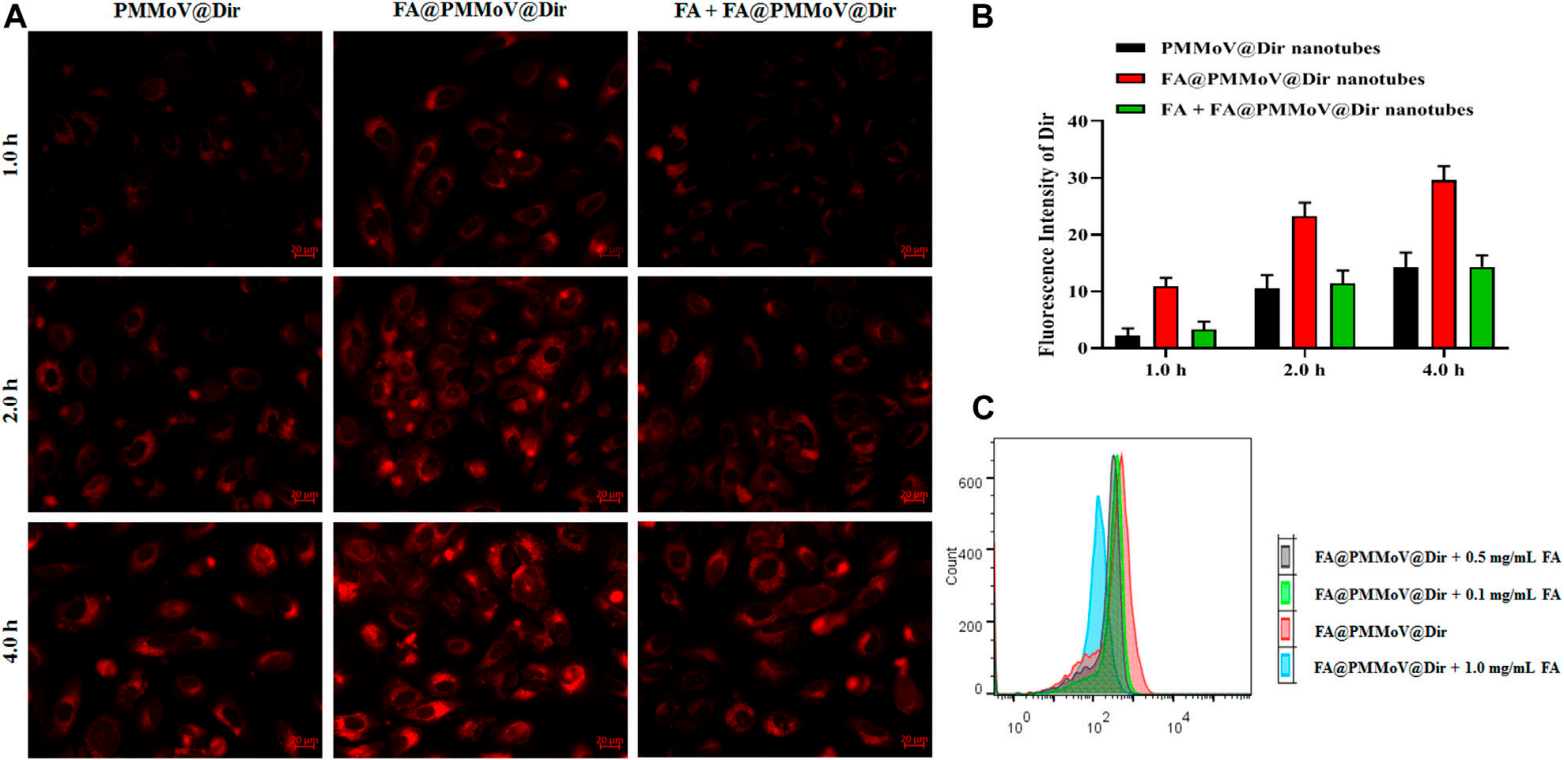
Uptake of FA@PMMoV nanotubes by tumor cells. **(A)** Images showing the fluorescence of FA@PMMoV@Dir nanotubes in MCF-7 cells, using PMMoV@Dir nanotubes as control and free FA as receptor competitor. **(B)** The semi-quantitative fluorescence signals from **(A)**. **(C)** The fluorescence intensity of FA@PMMoV@Dir nanotubes in MCF-7 cells treated with different concentrations of free FA (0.1, 0.5, and 1.0 mg/ml).

To investigate the internalization of FA@PMMoV nanotubes, fluorescent probe FA@PMMoV nanotubes labelled with Dir were prepared using the same method used to create FA@PMMoV@PTX. The internalization of FA@PMMoV nanotubes by MCF-7 cells was visualized under an inverted fluorescence microscope. As shown in Figures 3A,B, the fluorescence signals in cells treated with FA@PMMoV@Dir nanotubes was higher than in those treated with PMMoV@Dir nanotubes at various points in time, suggesting that the decorated FA increased the intracellular distribution of nanotubes in tumor cells due to their overexpression of folate receptors. To further verify the targeting mediation effect of FA on FA@PMMoV@NR nanotubes, free FA was added to competitively bind to the folate receptors highly expressed on tumor cells. Comparison of the fluorescence signals showed that the intracellular distribution of FA@PMMoV@Dir nanotubes was decreased after incubation with free FA, confirming the tumor-homing effect of FA. The intracellular fluorescent intensity was measured by flow cytometry and showed the similar results (Supplementary Figure 2). The competitive test was also performed by flow cytometry (Figure 3C), and the internalization of FA@PMMoV@Dir nanotubes was again reduced during incubation with free FA.

### *In vitro* Antitumor Activity

**FIGURE 4:**
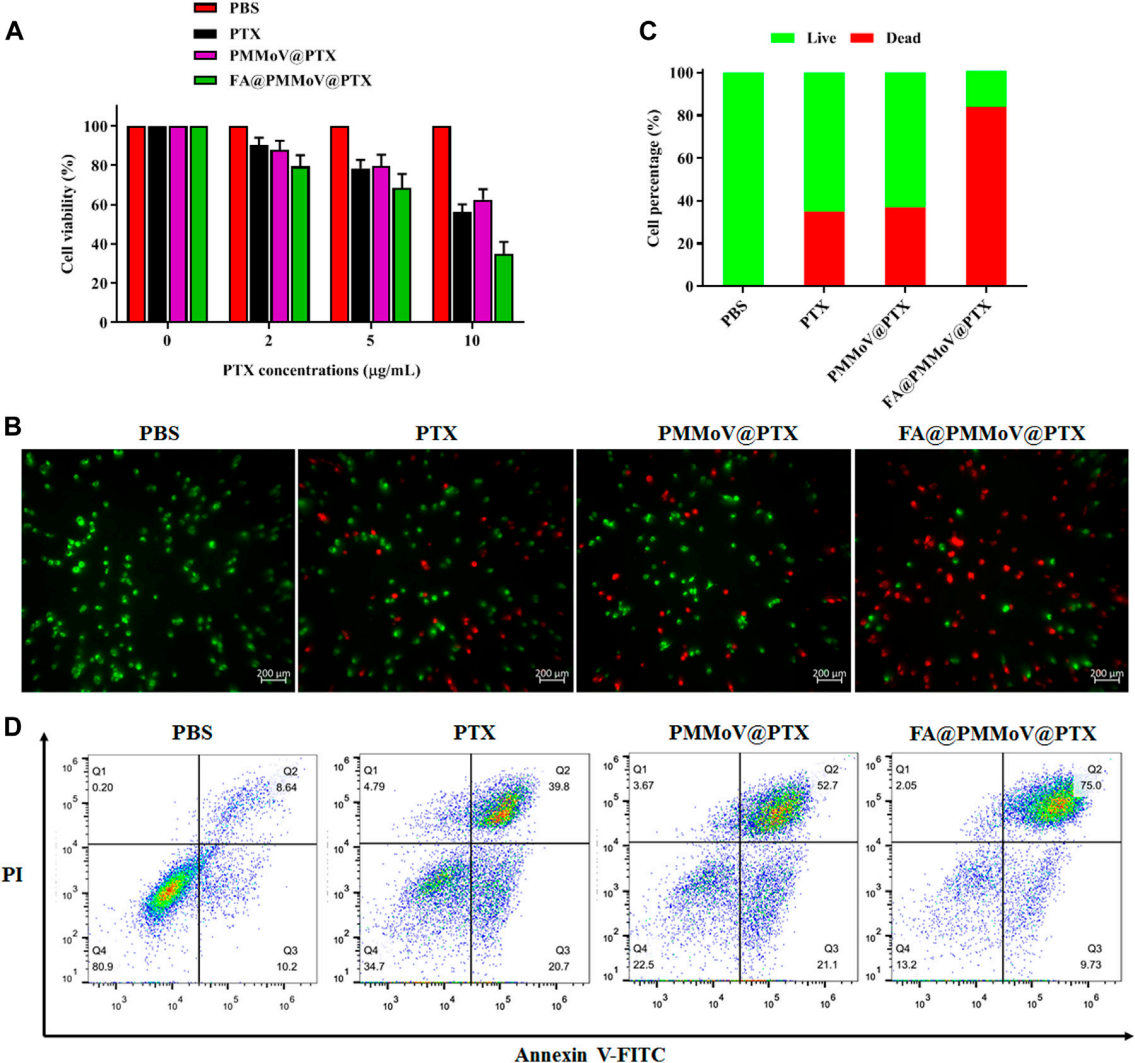
The *in vitro* anti-tumor activity of FA@PMMoV@Dir nanotubes. **(A)** Cell viability of MCF-7 treated with different concentrations of FA@PMMoV@Dir nanotubes. **(B)** Images showing the fluorescence of MCF-7 cells co-stained with calcein-AM and Ethd-1 and treated with FA@PMMoV@Dir nanotubes. **(C)** The semi-quantitative values of fluorescence signals from (B). **(D)** Flow-cytometry-based apoptosis assay for MCF-7 cells treated with FA@PMMoV@Dir nanotubes.

The *in vitro* anti-tumor activity of FA@PMMoV@PTX nanotubes was investigated using a CCK-8 assay (Jing et al., 2021). As shown in Figure 4A, FA@PMMoV@PTX nanotubes showed the best anti-tumor activity in comparison to that of free PTX and PMMoV@PTX nanotubes, benefiting from specific distribution and pH-triggered drug release of FA@PMMoV@PTX nanotubes. The liveanddead co-staining (Figure 4B) further proved that treatment with FA@PMMoV@PTX nanotubes provided the stronger anti-tumor activity. Annexin V/PI staining was used to assess the effects of FA@PMMoV@PTX nanotubes on apoptosis of MCF-7 cells. As shown in Figure 4C, FA@PMMoV@PTX nanotubes led to an obvious increase in numbers of apoptotic cells compared with controls treated with free PTX and PMMoV@PTX nanotubes.

## Conclusion

FA@PMMoV@PTX nanotubes were successfully prepared by PEG coprecipitation. There was good uptake of FA@PMMoV@PTX nanotubes by tumor cells because of the folate receptors overexpressed in them. Furthermore, the acid tumor microenvironment caused the degradation of FA@PMMoV@NR nanotubes, and further promoted the release of encapsulated PTX from nanotubes. The increased distribution and PTX release greatly enhanced the cytotoxicity of FA@PMMoV@NR nanotubes against MCF-7 cells in comparison to the commercial PTX. Although the *in vivo* efficacy of FA@PMMoV@NR nanotubes is unknown, the *in vitro* results suggest that PMMoV and other plant viruses are good potential candidates for drug delivery.

## Materials and Methods

### Preparation of FA@PMMoV@PTX Nanotubes

Genetic engineering techniques were used to form rod-shaped structures of nanoparticles based on PMMoV. The PMMoV vector was constructed and transformed into *Agrobacterium tumefaciens* (GV3101) which was delivered to *Nicotiana benthamiana* by infiltration. Successful infection was confirmed by RT-PCR and Western blot detections as described before (Han et al., 2020). Virus-infected leaf tissues (100 g) were harvested 28 days post infiltration and homogenized in 300 ml of extraction buffer (0.2 M sodium phosphate buffer; pH 7.2). After squeezing the slurry through preboiled cheesecloth the homogenate was emulsified in a mixture of 8% chloroform while being stirred at 25°C for 30 min. The mixture was centrifuged at 10,000 g for 30 min and the supernatant was collected. After addition of 8% PEG8000 (w/v) and 3% NaCl (w/v), the mixture was stirred gently for 1 h at 4°C, then kept at 4°C for 4 h and centrifuged at 8,000 g for 20 min. The resultant pellet was suspended in 50 ml of storage buffer (0.01 M sodium phosphate, pH 7.2) over night and clarified by centrifugation at 5,000 g for 10 min. To remove the PEG, the suspension was treated with 8% chloroform and centrifugated at 70,000 g for 4 h through a 25% sucrose cushion. The resulting pellet was suspended in 5 ml of storage buffer, providing a milky white suspension of purified virus which was stored at −80°C.

### Viral Protein Analysis

Systemic leaves of *Nicotiana benthamiana* (100 mg) were collected and ground in liquid nitrogen, then suspended in 200 µL of protein extraction buffer (50 mM sodium phosphate buffer pH 7.0, 5 mM β-mercaptoethanol, 10 mM EDTA, 0.1% Triton X-100). Crude extracts were mixed with 5×loading buffer then separated by SDS-PAGE. Western blotting analysis was performed to detect PMMoV infection with anti-PMMoV Coat protein (CP) antibody (Genscript).

### Loading Drug Combinations in the PMMoV Nanoparticles

Suspensions containing 100 mg of purified virus were mixed with the various combinations of powders (a. 2% FA-PEG, 8% PEG6000, 5% PTX; b. 2% FA-PEG, 8% PEG6000, 1% Dir; c. 2% FA-PEG, 8% PEG6000, 1% Nile red; d. 8% PEG6000, 1% Dir; e. 8% PEG6000, 5% PTX). The mixtures were kept in the dark overnight at 4°C. and then dialyzed against 0.1 M PBS for 12 h at 4°C using a dialysis bag (MWCO = 100 kDa). The post-dialysis fluids were freeze dried and stored at 4°C.

### Characterization of FA@PMMoV@PTX Nanotubes

FA@PMMoV@PTX nanotubes were dispersed in PBS with different pH values (1.0, 5.0, and 7.4) and then incubated for 24 h. After that, FA@PMMoV@PTX nanotubes (2.5 mg/ml) were sprayed onto 300-mesh copper grids, air-dried and negatively stained with 1% uranyl acetate solution for 1 min. After rinsing, the grids were observed under the transmission electron microscopy (Hitachi, H7650). The zeta potential and particle size of FA@PMMoV@PTX nanotubes (0.1 mg/ml) were determined by dynamic light scattering (Malvern, Zetasizer 3000HS).

The content of PTX in FA@PMMoV@PTX nanotubes was determined by HPLC after dispersal of the nanotubes in DMSO and passing through a 0.22 μm filter. The conditions were as follows: C18 (5 μm, 200 × 4.6 mm), mobile phase (acetonitrile: water, 60:40), detection wavelength 227 nm.

### pH-Triggered PTX Release of FA@PMMoV@PTX Nanotubes

The pH-triggered release behavior of PTX from FA@PMMoV@PTX nanotubes was investigated by the dialysis method in PBS (pH 1.0, 5.0, and 7.4) containing 1.0 M sodium salicylate. The free PTX (control) or FA@PMMoV@PTX nanotubes (equal amounts of PTX) were transferred into a dialysis bag (MWCO: 3.5 kDa), and horizontally shaken (100 rpm) at 37°C. At predetermined time intervals, the release medium outside of the dialysis bag was collected, and then replenished with fresh release medium. The samples were filtered through a 0.22 μm filter, and then PTX was assayed by HPLC. All drug release tests were performed three times.

To monitor pH-triggered drug release using fluorescence imaging, the fluorescent probe NR was encapsulated in FA@PMMoV nanotubes, using the same preparation method as for FA@PMMoV@PTX. The fluorescence of NR was quenched after encapsulation in FA@PMMoV nanotubes and was progressively recovered as NR was released from the nanotubes. 0.5 ml FA@PMMoV@Dir nanotubes were incubated with 0.5 ml PBS of different pH values at 37°C for 24 h. The fluorescent signals (Ex = 579 nm, Em = 610–640 nm) were detected using the *In-Vivo* imaging system (PerkinElmer, IVIS Lumia III).

### Cell Culture

The human breast cancer drug-sensitive cell MCF-7 was used in this study, and it was continuously cultured in 1,640 medium (containing 100 U/mL penicillin and streptomycin) containing 10% (v/v) fetal calf serum.

### Cellular Uptake

To investigate the internalization of FA@PMMoV nanotubes, fluorescent probe Dir was used to label FA@PMMoV nanotubes using the same method as for FA@PMMoV@PTX nanotubes. MCF-7 cells were seeded in 24-well plates at 1×10^4^ cells per well, and then incubated with FA@PMMoV@Dir nanotubes for 1.0, 2.0, or 4.0 h. PMMoV@Dir nanotubes were used as control, and free FA was used to competitively bind to folate receptors. After incubation, the cells were washed three times with cold PBS, fixed in 4% paraformaldehyde for 30 min, and then observed using an inverted fluorescent microscope (Zeiss, Axio Observer 5).

The competitive internalization of FA@PMMoV nanotubes by MCF-7 cells associated with folate receptor was investigated using flow cytometry (Beckman Coulter, CytoFlex S). MCF-7 cells were seeded in 6-well plates at 2×10^5^ cells per well, and then incubated with FA@PMMoV@Dir nanotubes and different concentrations of free FA (0.1, 0.5, and 1.0 mg/ml) for 2.0 h. Cells were then collected, washed with PBS and finally assayed using flow cytometry.

### Intracellular Drug Release

The intracellular drug release of FA@PMMoV nanotubes was investigated in MCF-7 cells, using human umbilical vein endothelial cells (HUVEC) as controls. Cells were seeded in 24-well plates at 1×10^4^ cells per well, and then incubated with FA@PMMoV@NR nanotubes for 24 h. After incubation, the cells were washed three times with cold PBS, fixed in 4% paraformaldehyde for 30 min, and then observed by laser scanning confocal microscopy.

### *In vitro* Anti-Tumor Activity

The *in vitro* anti-tumor activity of FA@PMMoV@PTX nanotubes was investigated using a CCK-8 assay. MCF-7 cells were seeded in 96-well plates at 1×10^3^ cells per well, and then incubated with different concentrations of FA@PMMoV@PTX nanotubes (PTX: 2, 5 and 10 μg/ml), using free PTX and PMMoV@PTX nanotubes as a control. After incubation for 24 h, 10 µL CCK-8 solution was added to each well for a further 2 h, and the absorbency was measured at 450 nm to calculate the survival rates.

The death rate of tumor cells effect was further verified by the LIVE/DEAD co-staining. (Invitrogen, United States) (Gallardo-Rivera et al., 2018). Cells were seeded in 12-well cell culture plates at a density of 2×10^4^ per well, and then incubated with FA@PMMoV@PTX nanotubes (PTX: 10 μg/ml), using free PTX and PMMoV@PTX nanotubes as a control. After incubation for 24 h, cells were washed with cold PBS, and then stained with calcein-AM (green) and EthD-1 (red) according to the manufacturer’s protocol (Invitrogen), followed by observation under an inverted fluorescence microscope.

### Apoptosis Detection *in vitro*

MCF-7 cells were seeded at a density of 1×10^5^ per well in 6-well, and then incubated with FA@PMMoV@PTX nanotubes for 24 h, using free PTX and PMMoV@PTX nanotubes as a control. Cells were then collected, resuspended in PBS, incubated with annexin V-FITC/propidium iodide, and analyzed by flow cytometry (Alsagaby et al., 2020).

### Statistical Analysis

Data were processed using SPSS 14 statistical software. Measurements were expressed as mean ± standard error, and statistical differences were tested by one-way ANOVA followed by the analysis using *t*-test and post hoc Fisher’s test after the homogeneity test of variance. The tested differences were considered statistically significant if *p*＜0.05.

**SCHEME 1:**
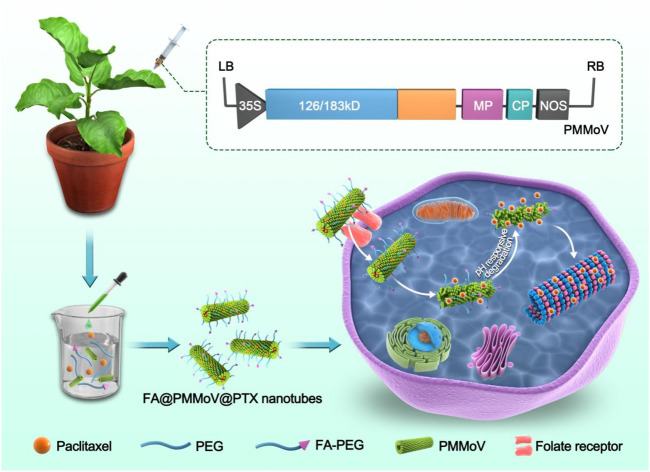
FA@PMMoV@PTX nanotubes were prepared by PEG coprecipitation. FA@PMMoV@PTX nanotubes were taken up well by tumor cells because of the folate receptors overexpressed in them. A pH-responsive drug release in the acid tumor microenvironment led to good delivery of PTX.

## Data Availability

The original contributions presented in the study are included in the article/Supplementary Material, further inquiries can be directed to the corresponding authors.

## Supplementary Material

Supplementary Figure 1 Synthesis and characterization of FA-PEG. (A) Synthesis route of FA-PEG. (B) ^*1*^ *H* NMR spectra of FA, PEG and FA-PEG.

Supplementary Figure 2 Intracellular fluorescence intensity, measured by flow cytometry, of MCF-7 cells ncubated with FA@PMMoV@Dir nanotubes for 2.0 h, PMMoV@Dir nanotubes and FA + FA@PMMoV@Dir nanotubes as control (n = 3, *P*<0.05).
