# Supplementary figures and images for: Tumor Microenvironment Responsive Pepper Mild Mottle Virus-Based Nanotubes for Targeted Delivery and Controlled Release of Paclitaxel
Source: Front Bioeng Biotechnol. 2021 Sep 30;9:763661. doi: 10.3389/fbioe.2021.763661 (PMC8514841; doi:10.3389/fbioe.2021.763661)

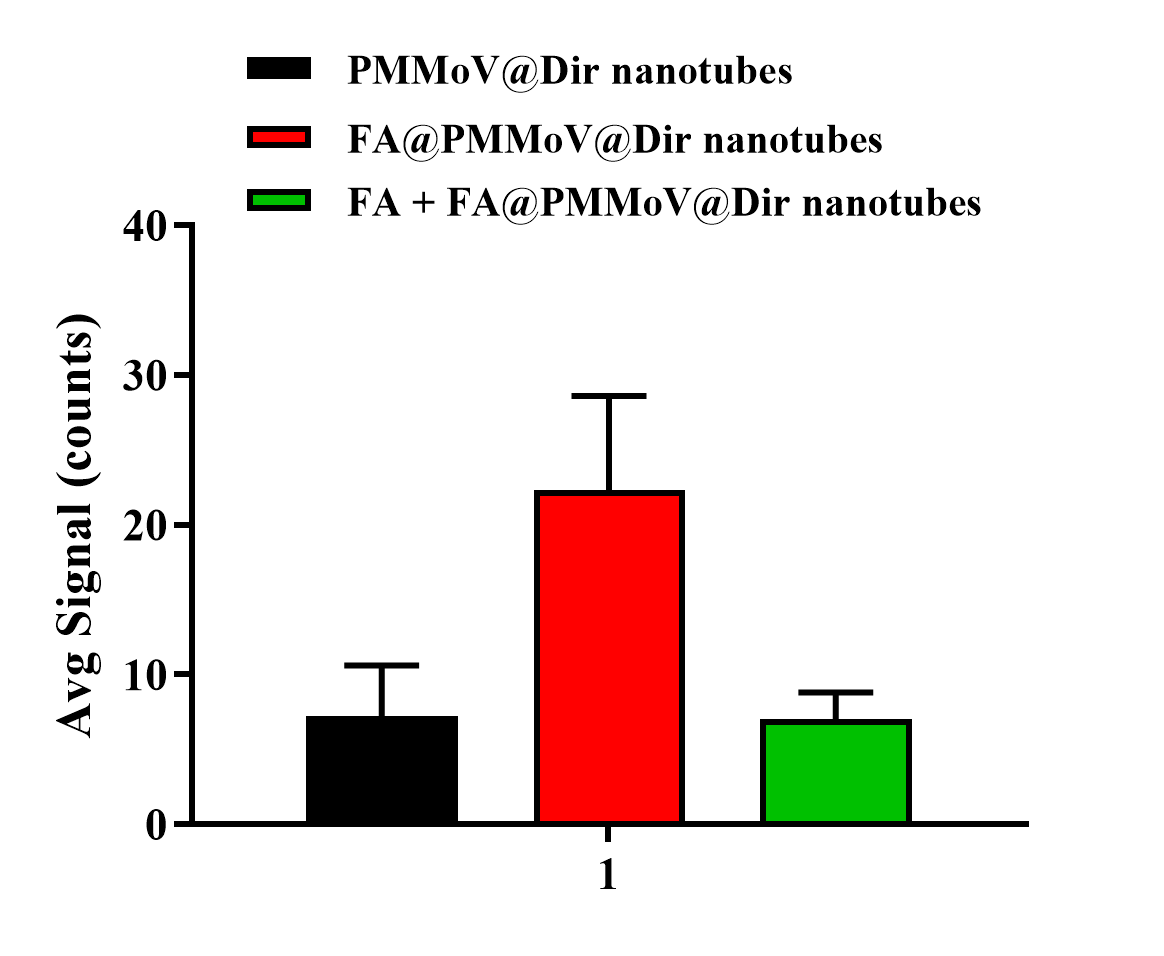

Supplement: Supplementary file 1 [file Image2.TIF]
